# Two latent classes of diagnostic and treatment procedures among traumatic brain injury inpatients

**DOI:** 10.1038/s41598-020-67576-4

**Published:** 2020-07-02

**Authors:** Hind A. Beydoun, Catherine Butt, May A. Beydoun, Shaker M. Eid, Alan B. Zonderman, Brick Johnstone

**Affiliations:** 1 Department of Research Programs, Fort Belvoir Community Hospital, 9300 DeWitt Loop, Fort Belvoir, VA 22060 USA; 2 Intrepid Spirit Center, Defense and Veterans Brain Injury Center, Fort Belvoir, VA USA; 3 Laboratory of Epidemiology and Population Sciences, National Institute On Aging, NIA/NIH/IRP, Baltimore, MD USA; 4 Department of Medicine, Johns Hopkins University School of Medicine, Baltimore, MD USA

**Keywords:** Diseases, Health care, Medical research, Neurology, Risk factors

## Abstract

To characterize latent classes of diagnostic and/or treatment procedures among hospitalized U.S. adults, 18–64 years, with primary diagnosis of TBI from 2004–2014 Nationwide Inpatient Samples, latent class analysis (LCA) was applied to 10 procedure groups and differences between latent classes on injury, patient, hospital and healthcare utilization outcome characteristics were modeled using multivariable regression. Using 266,586 eligible records, LCA resulted in two classes of hospitalizations, namely, class I (n = 217,988) (mostly non-surgical) and class II (n = 48,598) (mostly surgical). Whereas orthopedic procedures were equally likely among latent classes, skin-related, physical medicine and rehabilitation procedures as well as behavioral health procedures were more likely among class I, and other types of procedures were more likely among class II. Class II patients were more likely to have moderate-to-severe TBI, to be admitted on weekends, to urban, medium-to-large hospitals in Midwestern, Southern or Western regions, and less likely to be > 30 years, female or non-White. Class II patients were also less likely to be discharged home and necessitated longer hospital stays and greater hospitalization charges. Surgery appears to distinguish two classes of hospitalized patients with TBI with divergent healthcare needs, informing the planning of healthcare services in this target population.

## Introduction

Traumatic brain injury (TBI), a neurotrauma resulting from a mechanical force applied to the head, remains an issue of global health significance despite greater awareness, availability of guidelines and technological advancements in the realm of diagnosis and treatment of this complex condition^1–5^. Worldwide TBI incidence rate is estimated to range between < 100 and > 700 per 100,000 individuals, with variability in estimates attributed to differences in TBI conceptualization and operationalization^3^. In the United States, TBI affects approximately 1.7 million individuals, causing 50,000 deaths, 275,000 hospitalizations, and 70,000 individuals with long-term disability on an annual basis^6–8^. Economic losses attributed to TBI within the United States population have been estimated at $76.5 billion for the year 2000^9^. Recent estimates suggest direct costs of $9.2 billion, indirect costs of $51.2 billion through lost productivity and total medical costs ranging between $48.3 billion and $76.5 billion for the year 2013^8^. According to the Centers for Disease Control and Prevention (CDC) surveillance systems, whereas TBI-related deaths have declined, emergency department visits and hospitalizations linked to TBI have risen between 2001 and 2010^1^.

TBI presentation can range from mild alterations of consciousness to death^1^. Patients who experience TBI may have concomitant injuries (e.g. spinal cord injury) that need to be addressed and these injuries are often linked to the event that resulted in their TBI^10^. Consequently, TBI patients may receive a wide range of healthcare services^2^. On the other hand, TBI management within an acute care setting depends on injury severity, mechanism of injury and time since injury, with a general goal of homeostatic stabilization and prevention of secondary injuries^6^. As such, TBI can potentially manifest as a concussion, extra-axial hematomas, contusions, traumatic subarachnoid hemorrhage, and/or diffuse axonal injury, necessitating a wide range of diagnostic and/or treatment procedures within an acute care setting^1–3^. These surgical and non-surgical procedures have been previously classified as head elevation, hyperventilation, seizure prophylaxis, medically induced comatose state, therapeutic cooling, intracranial pressure monitoring, craniotomy and decompressive craniectomy^1^. Additional procedures include endotracheal intubation and mechanical ventilation, neurologic surgery to remove foreign matter or drain contusions and hematomas, as well as intensive care monitoring^6^. Novel TBI therapies cover the spectrum of injury severity as well as the continuum of care which includes rehabilitation for the improvement of independent functioning, social integration, and disability adaptation^6, 11^.

Although optimal management of TBI has been described in clinical guidelines and may have improved standard of care, it cannot be applied without taking individual circumstances into consideration^1, 2, 12, 13^. Given the multitude of concomitant procedures that may be applied to any given patient, an understanding of the patterns, predictors and outcomes of resource utilization among patients diagnosed with TBI is warranted. In particular, an examination of how distinct classes of diagnostic and/or treatment procedures tend to cluster may improve our understanding of healthcare needs attributed to TBI. The purpose of this cross-sectional study is to perform latent class analyses (LCA) in order to characterize clusters of diagnostic and/or treatment procedures among hospitalized U.S. adults, 18–64 years of age, with a primary diagnosis of TBI. As a methodology, LCA is often applied to examine associations between observed variables assuming the existence of clusters of unobserved categorical variabless. Although it has been applied extensively for the purpose of classification and identification of disease patterns, it has rarely been applied in the context of diagnostic and/or treatment procedures. In this study, the following research questions were addressed using the 2004–2014 Nationwide Inpatient Sample (NIS) databases:

1. What are the distinct latent classes of diagnostic/treatment procedures?
2. What are the predictors of latent classes of diagnostic/treatment procedures?
3. How do latent classes of diagnostic and/or treatment procedures predict discharge status, length of hospital stay and hospitalization charges?
4. Do healthcare utilization outcomes of these latent classes differ by sex, age, race/ethnicity, payer type or urban–rural location?

Based on similarly conducted studies^9, 15–19^, we hypothesized that utilization of healthcare resources in the context of TBI may be influenced by factors at different levels of organization, including injury, patient and hospital characteristics. We also hypothesized that socioeconomic disparities exist within the U.S. healthcare system in terms of utilization of healthcare resources pertaining to TBI. Exploring the clustering of distinct diagnostic/treatment procedures as well as the identification and characterization of a small number of diagnostic/treatment procedure classes can inform the planning of future healthcare services to improve outcomes among distinct patient groups that may experience TBI.

## Methods

### Data source

The Agency for Healthcare Research and Quality (AHRQ) Healthcare Cost and Utilization Project (HCUP) Nationwide Inpatient Sample (NIS) is the largest publicly available, all-payer inpatient care database of community hospitals in the United States. Each year, 5–8 million hospital discharge records are sampled using a 20% stratified sample of hospitals (before 2012) or hospital discharge records (since 2012) from all participating HCUP states, with strata defined based on the following hospital characteristics: ownership/control, bed size, teaching status, urban/rural location and U.S. region. Data elements within the NIS database include patient demographics, 15 or more diagnoses, 15 or more procedures, hospital course and outcomes. This retrospective study is based on a AHRQ project which was approved by an Institutional Review Board in accordance with principles outlined by the Declaration of Helsinki. This study received ethical approval through the Department of Research Programs at Fort Belvoir Community Hospital as research not involving human subjects. Because of its retrospective nature, no informed consent was obtained from subjects, parents and/or legal guardians for this study.

### Study population

Eligibility criteria were defined based on recently published TBI studies that have used the NIS database^9, 15–19^. The study population consists of hospitalization records from the 2004–2014 NIS databases that met the following inclusion criteria: (1) adults (18–64 years of age); (2) Clinical Classifications Software (CCS) code of 233 assigned by AHRQ for TBI; (3) Primary diagnosis of TBI using ICD-9-CM codes recommended by the CDC, whereby variable DX1 was coded as ‘fracture of cranial vault, skull base, or facial bone with intracranial injury’ (800.0–801.9, 803.0–804.9) or ‘concussion, cerebral contusion, subdural hematoma, epidural hematoma, other and unspecified traumatic intracranial hemorrhage’ (850–854.19). Hospitalization records were excluded from the study if they met at least one of the following criteria: (1) primary diagnosis of TBI history (V1552); (2) elective hospital admission; (3) Abbreviated Injury Severity (AIS) score deemed “unsurvivable”.

### Variable definitions

Using 15 procedure data elements, the 2004–2014 NIS database that consists of eligible hospital discharges was transposed from a wide to a long format to explore frequencies of ICD-9-CM procedure codes. Using the long database, a listing of ICD-9-CM procedure codes was generated and similar codes were combined into a limited number of procedure groups, taking frequencies into consideration. Within the wide database, an indicator variable was created to flag hospital records that utilized each of these procedure groups. Using LCA, two classes of diagnostic and/or treatment procedures were identified taking clustering of procedure groups into consideration.

Injury severity, patient and hospital-level characteristics were evaluated as predictors of procedure class. Furthermore, procedure classes were evaluated as predictors of selected healthcare utilization outcomes, namely, discharge status, length of hospital stay and hospitalization charges, before and after stratifying by selected characteristics. Injury severity among TBI-affected patients was calculated using the AIS. ICD-9-CM diagnostic codes within the NIS database were translated into AIS scores specific to the head and/or neck region using a freely available Stata program. The highest AIS score was chosen for categorizing injury severity as ranging from 1 (“minor”) to 6 (“unsurvivable”), and records with AIS of 6 were excluded. Subsequently, ‘mild’ TBI was defined among patients with head AIS score between 1 and 2, ‘moderate’ TBI among patients with head AIS score of 3 and ‘severe’ TBI among patients with head AIS between 4 and 5, as described elsewhere^9, 20^. Patient-level characteristics were defined as age, race/ethnicity, primary payer, as well as year, quarter and weekend admission. Hospital-level characteristics were defined as hospital region, control, urban–rural location, teaching status and bed size. Discharge status was defined as an ordinal variable, with the following categories: discharged home, discharged to institution or died. Length of hospital stay and hospitalization charges (‘U.S. dollars’, adjusted based on trends in 2004–2014 Consumer Price Index (https://www.in2013dollars.com/Hospital-services/price-inflation/2004-to-2014?amount=1) were defined as log_e_-transformed outcomes for the purpose of regression modeling.

### Statistical analysis

All statistical analyses were conducted using Stata release 15 (StataCorp, College Station, TX), taking complex sampling design into consideration. Descriptive statistics included mean (± standard error) for quantitative variables and frequencies with percentages for qualitative variables. Bivariate associations were examined using uncorrected Chi-square and design-based F-tests, as appropriate. Multiple linear, binary and multinomial logistic regression models were constructed to estimate beta (β) coefficients, odds ratios (OR) and relative risk ratios (RRR) with their 95% confidence intervals (CI). LCA was used as an exploratory, model-based technique of clustering, as previously described by Shahraz and colleagues in the context of diagnostic codes^21^. Specifically, LCA inputs observed procedure groups defined as dichotomous variables to predict procedure class membership. LCA-defined classes are latent constructs reflected by correlations among observed procedure groups^21^. Two outputs result from LCA, namely, probability of class membership for each observed procedure group and overall prevalence of hospital discharges within each class, whereby an Expectation Maximization Algorithm is used to generate class membership likelihood through an iterative process^21^. We used the *gsem* command in STATA to perform LCA and selected the number of distinct classes based on criteria of model fit and substantive interpretability^21^. Model fit was determined on the basis of Akaike Information Criterion and Bayesian Information Criterion, which led to deciding the appropriate number of latent classes. Posterior probabilities were estimated by using the Bayes theorem, and those were the same for all records with specific patterns of procedure groups. The higher these posterior probabilities the more the certainty of belonging to a specific class. Supplementary Methods S1 presents sample STATA code related to LCA. After evaluating the assumptions of missingness completely at random, we applied multiple imputation techniques with five datasets. Two-sided statistical tests were conducted and *p* < 0.05 was considered statistically significant.

## Results

Of 41,964,991 hospitalization records from the 2004–2014 NIS databases that corresponded to adult patients, 18–64 years of age, a total of 434,380 met all eligibility criteria with the exception of missing data on key variables. A total of 2,343 distinct ICD-9-CM procedure codes were identified of which 131 can be labeled as imaging procedures and 1,826 can be labeled as surgical procedures. When ICD-9-CM procedure codes were combined taking frequencies into consideration, a total of 23 procedure types were generated and later combined into 10 procedure groups. These procedure groups were defined as indicator (‘yes’ or ‘no’) variables and used to perform LCA. Prevalence rates of procedure types ranged between 1.4 per 1,000 records for hernia repair and 219.0 per 1,000 records for respiratory procedures (Table 1). Similarly, prevalence rates of procedure groups ranged between 34.0 per 1,000 records for health services that fall under miscellaneous surgeries and 266 per 1,000 records for health services that fall under ophthalmology, otorhinolaryngology and/or respiratory medicine (Table 2).

**Table 1:** Distribution of study-eligible hospital discharge records by 23 diagnostic and/or treatment procedure types: 2004–2014 nationwide inpatient sample (n = 434,380).

Procedure Type			Prevalence (95% CI) [per 1,000 hospital discharge records]
Serial #	ICD-9-CM codes	Description	
1	00.40–00.69 35.11–39.94 96.57 97.44 99.60–99.69	Cardiovascular surgery	136.0 (135.0, 137.0)
2*	00.94	Monitoring	1.6 (1.5, 1.8)
3	00.10–00.17 00.22–00.28 00.96	Drugs/Infusion	3.9 (3.8, 4.2)
4	01.01–03.02	Neurosurgery of the head	105.7 (104.8, 106.8)
5	03.09–03.99	Neurosurgery of the spine	27.5 (26.9, 27.9)
6	04.01–08.02	Neurosurgery of peripheral nervous system	6.7 (6.4, 6.9)
7	08.09–16.99 95.01–95.32 96.51 98.21	Ophthalmology	29.6 (29.1, 30.1)
8*	00.31–00.39 00.91–00.93 17.32–17.81 85.0–86.03	Computer Assisted Surgery/Breast Surgery/Other Surgery/Transplantation	2.4 (2.3, 2.6)
9	18.02–30.4 95.41–96.03 96.11 96.21 96.52–96.54 97.21 97.32–97.36 97.38–97.39 98.11–98.12 98.14 98.22 99.97	Otorhinolaryngology	49.5 (48.9, 50.2)
10	31.0–34.99 93.90–93.99 96.04–96.06 96.55–96.56 96.70–96.72 97.23 97.37 97.41 97.49 98.13 98.15	Respiratory	219.0 (217.8, 220.2)
11	39.95–41.99 99.00–99.59 99.71–99.79	Blood	115.4 (114.4, 116.4)
12	42.09–52.99 96.07–96.09 96.19 96.22–96.26 96.31–96.43 96.6 97.01–97.05 97.51–97.61 98.01–98.05	Gastroenterology	76.1 (75.3, 76.9)
13*	53.00–53.9 96.27	Hernia repair	1.4 (1.3, 1.5)
14	54.0–54.99 97.82–97.87 98.25	Abdominal surgery	15.0 (14.7, 15.4)
15	55.01–64.98 96.48–96.49 97.62–97.64 98.18–98.19 98.24 98.51 99.96	Urology	17.6 (17.2, 17.9)
16	65.09–75.7 96.14 97.71–97.75 98.17	Obstetrics and Gynecology	2.9 (2.8, 3.2)
17	00.70–00.87 76.01–84.99 97.11–97.14 98.26–98.29	Orthopedics/Knee/Hip surgery	149.9 (148.9, 151.0)
18	86.04–86.99 96.58–96.59 97.15–97.16	Skin	164.4 (163.3, 165.6)
19	00.01–0.09 87.01–88.98 92.02–92.19 92.21–92.39	Imaging/US/IVUS/Radiation	106.2 (105.2, 107.1)
20	89.01–91.93 93.01–93.09	Diagnostic tests	33.2 (32.6, 33.7)
21	93.11–93.89	Physical Medicine and Rehabilitation	46.9 (46.4, 47.6)
22	94.01–94.69	Behavioral Medicine	24.0 (23.6, 24.5)
23*	97.29 97.88–97.89 98.20 99.81–99.95 99.99	Other	4.6 (4.4, 4.8)

*CI* Confidence interval.

*Remove this procedure type from further consideration because of low prevalence rate.

**Table 2:** Distribution of study-eligible hospital discharge records by 10 diagnostic and/or treatment procedure groups: 2004–2014 Nationwide Inpatient Sample (n = 434,380).

Procedure types			Prevalence (95% CI) [per 1,000 hospital discharge records]
Group	Serial no.	Description	
A	1	Cardiovascular surgery	136.0 (135.0, 137.0)
B	4, 5, 6	Neurosurgery	133.9 (132.9, 134.9)
C	14–16	Surgery—Miscellaneous	34.0 (33.5, 34.6)
D	7, 9, 10	Ophthalmology, Otorhinolaryngology, Respiratory Medicine	266.0 (264.8, 267.4)
E	11	Blood	115.4 (114.4, 116.4)
F	12	Gastroenterology	76.1 (75.3, 76.9)
G	17	Orthopedics/Knee/Hip surgery	149.9 (148.9, 151.0)
H	18	Skin	164.4 (163.4, 165.6)
I	19, 20	Imaging/US/IVUS/Radiation/Diagnostic Tests	128.9 (127.8, 129.8)
J	21, 22	Physical Medicine and Rehabilitation/Behavioral Medicine	69.3 (68.5, 70.0)

*CI* Confidence Interval.

Overall, 266,586 records included 1+ of the procedure groups. Table 3 presents the results of the LCA using the 10 procedure groups (A–J). Of note, ICD-9-CM procedures labeled as hernia repair, computer assisted surgery/breast surgery/other surgery/transplantation, monitoring or other types of procedures were excluded from procedure groups because of sample size limitations. The LCA converged when two latent classes were specified, whereby 217,988 records belonged to Class I and 48,598 belonged to Class II.

**Table 3:** Latent class analysis of 10 diagnostic and/or treatment procedure groups: 2004–2014 Nationwide Inpatient Sample—2004–2014 Nationwide Inpatient Sample (n = 266,586).

Group/description	Class I (n = 217,988)			Class II (n = 48,598)		
	β	95% CI	*P*	β	95% CI	*P*
A. Cardiovascular surgery	− 2.21	− 2.24, − 2.19	< 0.0001	0.98	0.94, 1.01	< 0.0001
B. Neurosurgery	− 1.68	− 1.70, − 1.67	< 0.0001	− 0.10	− 0.12, − 0.081	0.63
C. Surgery: Miscellaneous	− 3.18	− 3.21, − 3.16	< 0.0001	− 1.97	− 2.00, − 1.94	< 0.0001
D. Ophthalmology, Otorhinolaryngology, Respiratory Medicine	− 0.72	− 0.73, − 0.71	< 0.0001	1.95	1.89, 2.00	< 0.0001
E. Blood	− 1.86	− 1.88, − 1.85	< 0.0001	− 0.39	− 0.41, − 0.36	< 0.0001
F. Gastroenterology	− 2.77	− 2.80, − 2.75	< 0.0001	− 0.44	− 0.46, − 0.42	< 0.0001
G. Orthopedics/Knee/Hip surgery	− 1.13	− 1.15, − 1.13	< 0.0001	− 1.07	− 1.09, − 1.05	< 0.0001
H. Skin	− 0.91	− 0.92, − 0.90	< 0.0001	− 1.41	− 1.44, − 1.38	< 0.0001
I. Imaging/US/IVUS/Radiation/Diagnostic Tests	− 1.38	− 1.38, − 1.36	< 0.0001	− 1.17	− 1.19, − 1.15	< 0.0001
J. Physical Medicine and Rehabilitation/Behavioral Medicine	− 1.91	− 1.92, − 1.89	< 0.0001	− 3.03	− 3.09, − 2.99	< 0.0001
Fit Statistics						
χ^2^, Sign	136,110.595, *P* < 0.0001					
AIC	2.501e+06					
BIC	2.501e+06					

*AIC* Akaike information criterion, *BIC* Bayesian information criterion.

With the majority of procedure groups appearing to load on Class I, we examined the prevalence of each procedure group by latent class. As displayed in Table 4, when each procedure group was entered as a predictor of latent class (II vs. I) in a logistic regression model, Class II records exhibited lower odds of undergoing ‘skin-related’ procedures (OR 0.53, 95% CI 0.52, 0.55) and/or ‘physical medicine, rehabilitation or behavioral health’ procedures (OR 0.31, 95% CI 0.30, 0.33) as compared to Class I, whereas Class I and Class II records had similar odds of receiving ‘orthopedics/knee/hip surgery’ (OR 1.04, 95% CI 1.02, 1.07) procedures. By contrast, all other procedure groups, including those involving surgery, were more frequently observed in the context of Class II versus Class I records.

**Table 4:** Prevalence rates of 10 diagnostic and/or treatment procedure groups by latent class: 2004–2014 Nationwide Inpatient Sample (n = 266,586).

Group	Description	Proportion (95% CI)		Class II versus I
		Class I (n = 217,988)	Class II (n = 48,598)	OR (95% CI)
A	Cardiovascular surgery	0.082 (0.081, 0.084)	0.846 (0.843, 0.849)	61.14 (59.39, 62.94)
B	Neurosurgery	0.155 (0.154, 0.157)	0.499 (0.495, 0.503)	5.39 (5.28, 5.51)
C	Surgery—Miscellaneous	0.039 (0.038, 0.0401)	0.129 (0.125, 0.131)	3.59 (3.47, 3.72)
D	Ophthalmology, Otorhinolaryngology, Respiratory Medicine	0.322 (0.319, 0.323)	0.936 (0.934, 0.938)	31.06 (29.91, 32.25)
E	Blood	0.135 (0.133, 0.136)	0.428 (0.423, 0.432)	4.81 (4.71, 4.92)
F	Gastroenterology	0.056 (0.055, 0.057)	0.428 (0.424, 0.433)	12.56 (12.25, 12.89)
G	Orthopedics/Knee/Hip surgery	0.243 (0.241, 0.244)	0.250 (0.246, 0.254)	1.04 (1.02, 1.07)
H	Skin	0.287 (0.286, 0.289)	0.178 (0.174, 0.181)	0.53 (0.52, 0.55)
I	Imaging/US/IVUS/Radiation/Diagnostic Tests	0.201 (0.200, 0.204)	.246 (0.242, 0.250)	1.29 (1.26, 1.32)
J	Physical Medicine and Rehabilitation/Behavioral Medicine	0.128 (0.127, .129)	0.044 (0.042, 0.046)	0.31 (0.30, 0.33)
Overall		0.803 (0.799, 0.806)	0.196 (0.193, 0.200)	–

*CI* Confidence interval.

Table 5 presents results of multivariable logistic regression models whereby injury, patient and hospital-level characteristics were entered simultaneously as predictors of latent classes of diagnostic and/or treatment procedures. Results suggested that Class II records were more likely than Class I records to belong to patients with moderate-to-severe TBI, admitted on weekends, to urban, medium-to-large hospitals located in the Midwestern, Southern or Western regions, and less likely to belong to female, non-White patients, who were > 30 years of age.

**Table 5:** Injury, patient and hospital-level predictors of latent classes of diagnostic/treatment procedures based on a two-class solution of latent class analysis: 2004–2014 Nationwide Inpatient Sample (n = 266,586).

	Proportion	Class II vs. Class I Log_e_ OR (95% CI)
**Injury characteristics**		
Mild	0.38	Ref
Moderate	0.38	1.32 (1.29, 1.36)
Severe	0.23	1.58 (1.55, 1.62)
**Patient characteristics**		
Sex		
Male	0.74	Ref
Female	0.26	− 0.089 (− 0.11, − 0.065)
Age (years)		
18–24	0.18	Ref
25–29	0.10	− 0.053 (− 0.09, − 0.013)
30–34	0.09	− 0.10 (− 0.14, − 0.060)
35–39	0.08	− 0.12 (− 0.16, − 0.076)
40–44	0.09	− 0.14 (− 0.18, − 0.10)
45–49	0.11	− 0.13 (− 0.17, − 0.09)
50–54	0.12	− 0.10 (− 0.14, − 0.066)
55–59	0.11	− 0.13 (− 0.16, − 0.09)
60–64	0.09	− 0.16 (− 0.20, − 0.12)
Race/ethnicity		
White	0.54	Ref
Black	0.11	− 0.053 (− 0.088, − 0.018)
Hispanic	0.11	− 0.001 (− 0.035, 0.033)
Other	0.06	0.027 (− 0.018, 0.071)
Unknown	0.18	− 0.10 (− 0.13, − 0.071)
Primary payer		
Medicare	0.12	Ref
Medicaid	0.18	0.38 (0.34, 0.42)
Private insurance	0.42	0.18 (0.14, .22)
Self-Pay	0.16	− 0.05 (− 0.09, − 0.01)
No charge	0.01	− 0.26 (− 0.37, − 0.16)
Other	0.10	0.08 (0.04, 0.13)
Year of admission		
2004	0.09	Ref
2005	0.07	0.11 (0.06, 0.17)
2006	0.09	0.12 (0.07, 0.17)
2007	0.08	0.11 (0.06, 0.15)
2008	0.08	0.08 (0.03, 1.13)
2009	0.08	0.12 (0.07, 0.17)
2010	0.11	0.05 (0.008, 0.10)
2011	0.09	0.07 (0.02, 0.12)
2012	0.09	− 0.033 (− 0.09, 0.03)
2013	0.09	0.12 (− 0.04, 0.30)
2014	0.09	0.06 (− 0.11, 0.23)
Admission quarter		
1st quarter	0.22	Ref
2nd quarter	0.26	− 0.050 (− 0.080, − 0.020)
3rd quarter	0.27	− 0.023 (− 0.053, 0.0058)
4th quarter	0.25	0.018 (− 0.012, 0.05)
Weekend admission status		
Monday–Friday	0.68	Ref
Saturday–Sunday	0.32	0.13 (0.11, 0.15)
**Hospital characteristics**		
Hospital region		
Northeast	0.19	Ref
Midwest	0.22	0.22 (0.18, 0.25)
South	0.36	0.31 (0.28, 0.34)
West	0.22	0.11 (0.08, 0.14)
Hospital control		
Government or private	0.56	Ref
Government, non-federal	0.04	0.17 (1.11, 1.24)
Private, not-for-profit	0.15	0.07 (0.02, 0.12)
Private, investor-owned	0.04	0.09 (0.03, 0.16)
Private	0.01	0.087 (− 0.060, 0.24)
Unknown	0.20	− 0.09 (− 0.26, 0.075)
Location and teaching status		
Rural	0.04	Ref
Urban—non-teaching	0.22	0.41 (0.33, .49)
Urban—teaching	0.73	0.59 (0.51, 0.68)
Hospital bed size		
Small	0.05	Ref
Medium	0.20	0.26 (0.21, 0.32)
Large	0.75	0.34 (0.29, 0.40)

*CI* Confidence interval, *RRR* relative risk ratio.

Table 6 presents latent classes of diagnostic and/or treatment procedures as predictors of discharge status, length of hospital stay and hospitalization charges. Overall, Class II patients were less likely to be discharged home and necessitated longer hospital stays and greater hospitalization charges than Class I patients. Significant interactions were observed between latent classes and selected socio-demographic characteristics in relation to healthcare utilization outcomes. Accordingly, stratified analyses were performed suggesting that disparities in healthcare utilization outcomes between latent classes may differ according to sex, age, race/ethnicity, payer type and urban–rural location.

**Table 6:** Latent classes of diagnostic and/or treatment procedures as predictors of discharge status, length of hospital stay and hospitalization charges: 2004–2014 Nationwide Inpatient Sample (n = 266,586).

	Discharge status*			Log_e_-length of stay*	Log_e_-hospital charges*
	Home [Ref.]	Institution Log_e_ RRR (95% CI)	Died Log_e_ RRR (95% CI)	*β* (95% CI)	*β* (95% CI)
Class II versus Class I					
Overall	–	1.73 (1.70, 1.76)	2.82 (2.77, 2.85)	0.98 (0.97, 0.99)	1.18 (1.17, 1.19)
Males	–	1.73 (1.69, 1.76)	2.78 (2.74, 2.83)	0.99 (0.98, 1.01)	1.19 (1.18, 1.20)
Females	–	1.76 (1.69, 1.81)	2.91 (2.83, 3.00)	0.98 (0.89, 0.94)	1.16 (1.14, 1.17)
< 50 years	–	1.75 (1.72, 1.79)	2.73 (2.67, 2.77)	1.01 (0.99, 1.02)	1.18 (1.17, 1.19)
≥ 50 years	–	1.69 (1.64, 1.74)	2.95 (2.87, 3.02)	0.93 (0.91, 0.95)	1.18 (1.16, 1.19)
White	–	1.77 (1.74, 1.82)	2.91 (2.84, 2.97)	0.97 (0.96, 0.98)	1.18 (1.17, 1.20)
Black/Hispanic/Other	–	1.63 (1.53, 1.68)	2.72 (2.64, 2.80)	1.04 (1.02, 1.06)	1.22 (1.20, 1.24)
Public insurance	–	1.52 (1.47, 1.57)	2.61 (2.53, 2.69)	0.99 (0.97, 1.01)	1.22 (1.20, 1.24)
Private insurance	–	1.84 (1.81, 1.87)	2.90 (2.85, 2.96)	0.97 (0.95, 0.98)	1.15 (1.14, 1.17)
Self-Pay/No charge/Other	–	1.69 (1.62, 1.78)	2.79 (2.65, 2.91)	0.99 (0.96, 1.02)	1.22 (1.19, 1.24)
Rural hospital	–	1.94 (1.74, 2.14)	3.22 (2.94, 3.51)	0.86 (0.79, 0.93)	1.16 (1.10, 1.22)
Urban hospital	–	1.73 (1.70, 1.76)	2.81 (2.76, 2.84)	0.98 (0.97, 0.99)	1.18 (1.17, 1.19)

*All models were adjusted for injury, patient and hospital characteristics.

*CI* Confidence interval, *RRR* relative risk ratio.

## Discussion

The heterogeneous nature of TBI and the virtual nonexistence of an “average” TBI patient have prompted the search for novel diagnostic tools including biomarkers as well as hindered the approval of safe and effective therapies by the U.S. Food and Drug Administration^2, 5^. Indeed, TBI exhibits a complex pathogenesis whereby a primary injury directly linked to external brain impact is followed by a secondary injury characterized by molecular, chemical, and inflammatory cascades that frequently occur from minutes to days after the occurrence of a primary injury^1, 2, 11^. Long-term physical, cognitive, and psychological sequelae associated with TBI may adversely affect the social and/or work functioning of TBI survivors for months to years after hospital discharge, often requiring prolonged rehabilitation^3, 4, 8, 11, 13^ and potentially leading to neurodegenerative disorders^2, 11^. As such, TBI is no longer perceived merely as an acute event but rather as a progressive injury and/or chronic disease which may manifest over hours, days, weeks, months or even years^2^.

In this cross-sectional study, we performed LCA to evaluate diagnostic and/or treatment procedures that tend to cluster within hospitalized TBI patients. The LCA identified two classes of records. Class I records likely correspond to patients who underwent mostly “non-surgical” procedures, whereas Class II records likely correspond to patients who underwent mostly “surgical” procedures, and clustering of procedure groups was predominantly driven by factors related to injury severity. In fact, procedures that are needed in an acute care setting immediately after occurrence of a TBI event were more prevalent among Class II versus Class I records, whereas procedures that are generally received by patients who no longer need stabilization were more frequently observed among Class I versus Class II records. Given the nature of these latent classes, their distribution according to injury, patient and hospital-level characteristics as well as healthcare utilization outcomes were as expected. For instance, Class II patients were more likely to have experienced moderate-to-severe injuries and, as a result, to have worse healthcare utilization outcomes than Class I patients. Results also suggested that Class II patients were more likely than Class I patients to receive healthcare services at medium-to-large urban hospitals with more resources for acute or intensive care. Disparities in healthcare utilization outcomes when comparing Class I versus Class II across socio-demographic factors may suggest that vulnerable populations are more likely to experience adverse events as a result of their injuries, irrespective of their healthcare needs.

Previous studies of TBI-related hospitalizations using large databases have similarly evaluated risk factors for healthcare utilization outcomes with an emphasis on the role played by TBI comorbidities as well as socio-demographic characteristics. In a retrospective cohort study, Brandel et al*.* used California Office of Statewide Health Planning and Development data to examine whether a comorbid psychiatric disorder was associated with a change in outcome among patients diagnosed with traumatic subdural hemorrhage^16^. Their results suggested that, depression (OR 0.64), bipolar disorder (OR 0.45), and anxiety (OR 0.37) were associated with reduced mortality, whereas psychosis (OR 2.12) and schizophrenia (OR 2.60) were associated with increased and anxiety was associated with reduced (OR 0.73) adverse discharge during a TBI hospitalization^16^. Asemota et al*.* applied multivariable logistic regression analyses to 2005–2010 NIS records and evaluated disparities in access to rehabilitative services according to race and insurance coverage^20^. Their results suggested reduced access to rehabilitation among racial and ethnic minorities, irrespective of insurance coverage, as well as reduced access to rehabilitation among uninsured populations, regardless of race/ethnicity^20^.

To date, a limited number of studies have attempted to identify clusters of ICD codes using LCA, and many of these studies were focused on psychiatric conditions^22–28^. For instance, Weich and colleagues evaluated the extent, nature and patterning of psychiatric co-morbidity using a representative sample of 7,325 individuals, 16 years and older, from the 2007 Adult Psychiatric Morbidity Survey^28^. LCA of 15 common mental health and behavioral problems resulted in a four-class model whereby 81.6% were classified as 'Unaffected', 12.4% as 'Co-thymia', 5.0% as 'Highly Co-morbid' and 1.0% as 'Addictions'^28^. Similarly, Liu and colleagues analyzed data on 430,569 patients from the Pennsylvania Health Care Cost Containment Council dataset (2000–2014) to identify opioid-related hospitalizations using primary and/or secondary ICD-9-CM hospital discharge codes for opioid use disorder (OUD), opioid poisoning, and heroin poisoning^25^. When LCA was applied to sociodemographic characteristics, pregnancy, alcohol, tobacco, substance use, and psychiatric disorders, five latent class groups were identified: “pregnant women with OUD”; “women over 65 with opioid overdose”; “OUD, polysubstance use and co-occurring psychiatric disorders”; “patients with opioid overdose without co-occurring polysubstance use”; “African American patients with OUD and co-occurring cocaine use”^25^.

TBI treatment modalities vary substantially according to injury severity and can range from daily cognitive therapy sessions to bilateral decompressive craniectomies^1^. This study found that the majority of adult patients who were hospitalized for TBI were more likely to have received services focused on non-surgical procedures. These procedures are often aimed at cultivating independent functioning, social integration, and disability adaptation^6^. This finding is consistent with the idea that mild TBI which may not require invasive procedures accounts for > 85% of all cases of TBI^29^. A systematic review of the literature by Wiart and colleagues identified 98 articles, including 15 controlled studies, focused on non-pharmacological treatment of psychological and behavioral disorders following TBI^13^. They concluded that whereas a holistic approach structured into programs, cognitive-behavioral therapy, as well as family/systemic therapy were recommended at all stages of TBI, relational and adaptive approaches, rehabilitation and vocational approaches, and psychoanalytical therapies may be useful, assuming that therapists were familiar with and trained in TBI^13^.

Study findings should be interpreted with caution and in light of several limitations. First, we relied on an administrative database consisting of patient- and hospital-level data elements that are typical of hospital discharge records which has limited information on physical examinations, laboratory tests and medications. In the absence of details pertaining to clinical presentation or reasons for hospital admission, we could not clearly distinguish hospitalizations resulting from TBI alone versus hospitalizations resulting from multiple traumas. Second, data clustering as a consequence of patient re-admission to one of the participating hospitals cannot be evaluated without access to unique patient identifiers. Third, many study variables, including TBI diagnosis, injury severity and procedures, were defined based on ICD-9 codes, potentially leading to misclassification bias. Specifically, TBI severity is usually classified into mild, moderate, and severe subtypes not on the basis of AIS, but rather on the basis of Glasgow Coma Scale (GCS) scores, duration of loss of consciousness (LOC) and duration of post-traumatic amnesia (PTA)^30^. Also, the determination of head AIS is quite difficult in clinical practice requiring extensive training of healthcare professionals at trauma centers. Although previously adopted by HCUP researchers, the AIS may not be reliably calculated from ICD-9-CM diagnostic codes in the context of head trauma related injuries. Non-differential misclassification of AIS may have resulted in an under-estimated association between AIS and procedure classes. Fourth, residual confounding by unmeasured or inadequately measured covariates may have led to biased measures of association. Fifth, this study design does not allow the establishment of temporality or causal relationships between exposure and outcome variables. Sixth, reliance on AIC and BIC can be considered a data-driven approach to choosing the number of classes and can potentially lead to overfitting. In this study, however, latent class models using three or more classes did not converge. Accordingly, the latent class analysis with two classes was the only option. Furthermore, the large sample size may have compensated for this data-driven approach. Finally, study results can only be generalized to U.S. hospitalized patients, whose characteristics may differ from those who sought outpatient care. Also, these results may be fairly specific to the U.S. healthcare system, which differs in many respects from other westernized countries, not the least by injury epidemiology.

In conclusion, hospitalized patients with TBI tend to fall in mostly “non-surgical” or “surgical” classes of diagnostic and/or treatment procedures, although orthopedic procedures appear to be common to both classes, with the latter being more severely injured and therefore requiring immediate attention. This classification may be useful for planning of healthcare services in the context of hospitalized patients with TBI by informing healthcare providers about healthcare needs of high-risk populations, especially in the context of sudden staff overload in emergency situations. Furthermore, predictive models linking these latent classes to healthcare utilization outcomes may aid healthcare professionals in clinical decision-making. Due to the exploratory nature of our analyses and the complexity of the TBI condition, labeling a new patient as belonging to one of the two clusters and consecutively predicting their clinical course may be difficult. By contrast, prediction of clinical outcome using machine learning may be more efficient in the context of databases whereby more detailed clinical characteristics are available. As such, prospective cohort studies are needed to confirm these exploratory findings.

## Supplementary information

Supplementary information 1

## Data Availability

The data that support the findings of this study are available from the Agency for Healthcare Research and Quality but restrictions apply to the availability of these data, which were used under license for the current study, and so are not publicly available. Data are however available from the authors upon reasonable request and with permission of Agency for Healthcare Research and Quality.
