# Supplementary material for: Two latent classes of diagnostic and treatment procedures among traumatic brain injury inpatients
Source: Sci Rep. 2020 Jul 2;10:10825. doi: 10.1038/s41598-020-67576-4 (PMC7331666; doi:10.1038/s41598-020-67576-4)
Supplement: Supplementary file 1 — Supplementary information 1 [file 41598_2020_67576_MOESM1_ESM.docx]

**Two Latent Classes of Diagnostic and Treatment Procedures among Traumatic Brain Injury Inpatients**

Hind A. Beydoun, PhD, MPH^;^ Catherine Butt, MA; May A. Beydoun, PhD, MPH; Shaker M. Eid, MD, MBA; Alan B. Zonderman, PhD; Brick Johnstone, PhD

**Supplementary Methods S1:**

**Sample STATA code for Latent Class Analysis**

*gsem (VAR1 VAR2 VAR3 VAR4 VAR5 VAR6 VAR7 VAR8 VAR9 VAR10 <- _cons), family(binomial) link(logit) lclass(A 2)*

*estat lcprob*

*estat lcmean*

*estat lcgof*

*predict cpost*, classposteriorpr*

*egen max = rowmax(cpost*)*

*generate predclass = 1 if cpost1==max*

*replace predclass = 2 if cpost2==max*

*tabulate predclass*
